# Incidence of Tetracycline and Erythromycin Resistance in Meat-Associated Bacteria: Impact of Different Livestock Management Strategies

**DOI:** 10.3390/microorganisms9102111

**Published:** 2021-10-07

**Authors:** Cecilia Fontana, Vania Patrone, Constanza Maria Lopez, Lorenzo Morelli, Annalisa Rebecchi

**Affiliations:** 1 Biotechnology Research Centre (CRB), Università Cattolica del Sacro Cuore, Via Milano 24, 2610 Cremona, Italy; cecilia.fontana@unicatt.it (C.F.); constanzamaria.lopez@unicatt.it (C.M.L.); lorenzo.morelli@unicatt.it (L.M.); 2 INTA EEA Famaillá, Tucumán 4172, Argentina; 3 DISTAS, Università Cattolica del Sacro Cuore, Via Emilia Parmense 84, 29100 Piacenza, Italy; vania.patrone@unicatt.it

**Keywords:** antibiotic resistance, lactic acid bacteria, coagulase negative staphylococci, animal husbandry, pork and llama meat

## Abstract

The extensive use of antibiotics as growth promoters, or their continued abusive misuse to cure or prevent the onset of bacterial infections as occurs in the intensive farming, may have played a pivotal role in the spread of reservoirs of antibiotic resistance (AR) among food-associated bacteria including pathogens representing risks to human health. The present study compares the incidence of tetracycline and erythromycin resistances in lactic acid bacteria (LAB) and coagulase negative staphylococci (CNS) from fermented products manufacturing using meat from intensive animal husbandry (industrialized manufacturing Italian salami) and from extensive farms (artisanal sausages facilities pork and llama Argentinean sausages). A higher incidence of tetracycline resistance (TET-R) compared to erythromycin resistance (ERY-R) was observed among the 205 isolates. Unlike CNS strains, the LAB showed a significant correlation between the TET-R and the ERY-R phenotypes. Genotypic assessment shows a high correlation with *tet*K and *tet*M for the TET-R strains and with *erm*B and *erm*C for the ERY-R strains. Multiple correspondence analyses have highlighted the association between AR phenotypes and CNS species isolated from Italian salami, while the susceptible phenotypes were associated with the LAB species from Argentinean sausages. Since antibiotic resistance in meat-associated bacteria is a very complex phenomenon, the assessment of bacterial resistance in different environmental contexts with diverse farming practices and food production technologies will help in monitoring the factors influencing AR emergence and spread in animal production.

## 1. Introduction

Intrinsic and acquired resistance to antibiotics have been described among bacteria; the former has minimal potential for horizontal spread. Acquired resistance, however, as a consequence of the acquisition of genetic material, such as a plasmid or a transposon coding for antibiotic-resistant genes, is potentially transferable, which represents a risk of spreading from one bacterium to another through several mechanisms [1].

Many studies have reported acquired resistance to erythromycin and tetracycline among lactic acid bacteria (LAB) and coagulase negative staphylococci (CNS), and therefore, their corresponding genetic determinants are considered an important potential cause of the horizontal spread of antibiotic-resistant (AR) genes between different bacterial species [2,3].

The improper use of antimicrobial drugs in animal husbandry—mainly tetracycline and erythromycin—has contribute to an increase in AR bacteria in animal food products [4,5]. Gevers et al. [6] reported a high incidence of tetracycline-resistant LAB in different types of commercial modified atmosphere-packed fermented dry Belgian sausages and showed that this population was dominated by *Latilactobacillus sakei*, *Companilactobacillus alimentarius* and *Lactiplantibacillus plantarum* species [7]. An analysis of slightly fermented Spanish sausages (chorizo, fuet and salchichon) revealed low percentages of tetracycline and chloramphenicol-resistant isolates of *L. sakei*, *Latilactobacillus* *curvatus* and *Leuconostoc mesenteroides* [8], while high frequencies of resistant isolates were found against gentamicin and ampicillin. Low levels of resistance to tetracycline and clindamycin were detected in *Lactobacillus* spp. From dry-cured Spanish sausages as well [9]. Lactobacilli from a traditional Italian salami (Ciauscolo) displayed a high level of resistance to aminoglycosides such as streptomycin and gentamicin, while resistance to tetracycline varied according to the species [10]. Zonenschain et al. [11] reported high erythromycin resistance among lactobacilli isolated from Italian sausages. On the other hand, in fermented llama meat sausages produced in Argentina, *L. sakei* showed high susceptibility to erythromycin, tetracycline, clindamycin, gentamicin and ampicillin [12]. 

With regard to antibiotic resistance genes, the most commonly detected in LAB isolates recovered from fermented sausages are *tet*M for tetracycline and *erm*B for erythromycin resistance [10,11,13]. 

Several studies regarding CNS have reported the occurrence of AR strains among starter cultures associated with fermented foodstuffs including meat, namely *S. carnosus* and *S. xylosus*. Marty et al. [14] investigated the AR of strains from traditionally fermented Swiss sausages. Resistance to penicillin and tetracycline was common in *S. xylosus* and was associated with the occurrence of *blaZ* and *tetK* genes, respectively; the species displaying the lower levels of resistance were *S. carnosus* and *S. equorum* [14]. Nunes et al. [15] detected resistance to vancomycin and to methicillin in 47% and 53%, respectively, of CNS isolated from Brazilian salami, including *S. xylosus*, *Staphylococcus succinus*, and *S. saprophyticus*. Simeoni et al. [16] reported the prevalence of *tetK*, *tetM* and *ermC* genes in resistant staphylococci isolated from the whole swine production chain in Italy. Rebecchi et al. [17] reported the high frequency of tetracycline and erythromycin resistance in CNS isolated from Italian salami production chain, with *erm*C, *tet*K and *tet*L as the most frequently detected AR genes. However, the authors found a strong reduction in AR strains in the final product, suggesting that the manufacturing process may contribute to the reduction in AR bacteria. Very recently, the phenotypic and genotypic AR profiles of CNS from Argentinean artisanal llama sausages were investigated [18]; as a result, a high incidence of resistance to oxacillin, ampicillin and erythromycin followed by tetracycline, kanamycin and gentamicin was shown. *BlaZ* and *tetK* were found in all the ampicillin-resistant and tetracycline-resistant strains, respectively, while the *mec*A gene was only detected in half of oxacillin-resistant CNS.

As summarized in the previous sentences, AR LAB and CNS have been found to be present in processed meat, with some differences tentatively related to the production technology, ripening period, the origin and kind of meat, the geographical location and the management of the farm. In this context, as stated by the FAO [19], intensive farming practices may have an important impact on the increase in resistance to antimicrobials since they have been associated as prophylaxis against infectious diseases, often for prolonged periods and for large populations of animals. On the contrary, extensive livestock farming systems, characterized by low productivity per animal and per surface, appear to only use antimicrobials for the treatment of infected or sick animals rather than for disease prevention or growth promotion. In addition to different livestock management strategies, the safety characteristics of the final microbiota of a fermented meat product can be influenced by other different factors, such as the microbial contamination of raw meat, the ingredients used, and the processing technology [20,21]. The aim of this present study was therefore to investigate the incidence and spread of tetracycline and erythromycin resistance in LAB, and CNS species isolated from meat products coming from two contrasting environments: Italy with intensive breeding practices and industrialized manufacturing; and Argentina with rearing extensive and artisanal sausages facilities. For this purpose, a very peculiar family of products such as those manufactured with llama meat were also included. The potential correlations between the occurrence of AR bacteria in these food items and their country of origin, animal husbandry management as well as the manufacturing practices were also investigated. 

## 2. Material and Methods

### 2.1. Bacterial Strains and Culture Conditions

A total of 121 LAB and 84 CNS strains included in this study were previously isolated from Italian Piacentino Salami DOP [11,22] and from fermented pork and llama meat produced in Argentina [12,18,23]. 

The strains were typified by randomly amplified polymorphic DNA (RAPD) technique and taxonomically identified by means of 16S RNA gene sequencing as belonging to *Latilactobacillus (L.) sakei, Lactiplantibacillus (L.) plantarum, Staphylococcus xylosus, Staphylococcus equorum* and *Staphylococcus saprophyticus* [13,14,20,24,25]. The Italian products were manufactured using meat from intensive swine production facilities, while the Argentinean fermented sausages were produced employing pork and llama meat obtained from animals raised on small rural-hub farms. Moreover, the llama breeding activities were located at 4320 m above sea level in the province of Jujuy, northwestern Argentina, under conditions of extensive pasture characterized by the grassland varieties typical of the Andean region and without any other breeding activity in the area.

The Italian and Argentinean sausages were produced without the use of starter cultures and their respective formulations and production processes were described by Zonenschain et al. [11] and Fontana et al. [12,23], respectively. Briefly, the Italian sausages were prepared according to the traditional technique using pork meat (75%) and lard (25%), salt (2.5%), black pepper (0.4%), white wine (0.5%), crushed garlic (0.2%), nitrate and ascorbic acid. The Argentinean pork and llama sausages were prepared mixing pork meat (85%) or llama meat (75% and 71.5%), respectively, with pork fat (between 10% and 25%), sodium chloride (2.6% and 2.4%), sugarcane (0.4% and 1.0%), binding and flavoring additives and nitrite/nitrate. 

*L. sakei* and *L. plantarum* strains were grown at 37 °C in a CO_2_-enriched atmosphere on an MRS (Oxoid, Italy) medium. The *Staphylococcus* (*S*) strains were cultured in BHI (Brain Heart Infusion, Oxoid) medium under aerobic conditions. Bacterial strains were stored at −80 °C in the appropriate broth supplemented with 20% glycerol [26].

### 2.2. Antibiotic Resistance and Determination of the Minimum Inhibitory Concentration 

Resistance to relevant antibiotics has been already demonstrated for the bacterial strains under investigation as previously reported [11,12,18,22]. In this study, the same reference methods were used to assess the susceptibility to tetracycline and erythromycin of all *L. sakei* and *L. plantarum* and CNS isolates, in order to ensure a uniform approach and allow data comparison. The minimum inhibitory concentration (MIC) (µg/mL) of the tested antibiotics was reached by the broth microdilution test method using a U-bottom 96-well microtiter plate recommended by the International Organization of Standardization/International Dairy Federation ISO10932/IDF233 standard [24] (for LAB) and CLSI [25] (for *Staphylococcus* spp.). Briefly, the bacterial inocula were prepared by suspending a single bacteria colony in 3 mL sterile 0.85% NaCl and adjusting the turbidity of the cells’ suspension to 1 (for *L. sakei* and *L. plantarum*) and 0.5 (for *Staphylococcus*) with McFarland standard equivalents. The bacterial suspension was diluted 500 and 100 times for *L. sakei* and *L. plantarum,* and staphylococci, respectively, in the recommended culture media. LSM broth (90% w/v Iso-sensitest broth and 10% MRS) pH 6.7 [26] was used for *L. sakei* and *L. plantarum*, while a cation-adjusted Mueller-Hinton broth (CAMHB) was used for the CNS isolates. The different antibiotics’ concentration ranges are shown in Table 1. 

Tetracycline and erythromycin (Sigma, St. Louis, MO, USA) were diluted in a LSM or CAMHB medium in the appropriate concentration. Fifty microliters of the two-fold concentration of antibiotic solutions were dispensed into a well and the microplates were subsequently inoculated with 50 µL of the cell suspension and incubated for 48 h at 30 °C under anaerobic conditions (Anaerocult, Darmstadt, Germany) for *L. sakei* and *L. plantarum*, and for 24 h at 37 °C under aerobic conditions for *Staphylococcus*. All experiments were performed in duplicate. The bacterial growth was measured as the cell density in the well bottom and compared to the growth in the control well containing the growth medium without antibiotics. *L. plantarum* LMG6907 and *S. aureus* ATCC29213 were used as quality control strains. The MIC value was defined as the lowest concentration of antibiotic with no visible growth in the microtiter well. Results of the tetracycline and erythromycin susceptibility assessment were interpreted according to the guidelines of the European Food Safety Authority [27] for *L. sakei* and *L. plantarum,* and CLSI [25] for *Staphylococcus*. The MIC breakpoints are reported in Table 1.

### 2.3. Detection of Antimicrobial Resistance Genes

DNA extraction from bacterial cultures was performed using the Microlysis solution (LABOGEN, UK) according to the protocol described by the manufacturer. The PCR amplification of the structural genes associated with TET and ERY resistance was performed using the primers and conditions listed in Table S1.

### 2.4. Statistical Analyses

Pearson’s correlation was used to analyze the association between phenotypic and genotypic features; *p*-values < 0.05 were considered statistically significant.

A contingency table was used to evaluate the relationship between the phenotypic antibiotic resistance (MIC values) and gene incidence. The Chi-square test (*p* < 0.05) was performed, and Pearson’s contingency coefficient was calculated to determine the weight and the significance of the association.

A logistic regression model was used to evaluate the association of TET and ERY phenotypic resistance with the country of origin of the strains (Italy/Argentina); the type of meat (pork/llama); and the bacterial group (LAB/CNS). The odds ratio (95% confidence interval) and the Wald ratio for the associated coefficients were calculated and a Chi-square test was performed to determine the differences between the odds of antibiotic resistance. Statistical analysis and data representation were performed using the XLSTAT software (Addinsoft Corporation, Paris, France, https://www.xlstat.com (accessed on 9 July 2021)) and INFOSTAT software (v. 2015).

## 3. Results and Discussion

### 3.1. Incidence of Tetracycline and Erythromycin Resistance 

The extensive use of antibiotics as growth promoters in animal husbandry for several decades until it was banned, in addition to the continued abusive misuse of tetracycline and erythromycin to cure or prevent the onset of bacterial infections, have played a pivotal role in the spread of silent reservoirs of antibiotic resistance among food-associated bacteria with their consequent risk to human health [28,29]. 

In this study, phenotypic and genotypic evaluations of tetracycline and erythromycin resistance were performed in LAB and CNS species. Despite the distant origins as well as the different production technologies and kinds of meat (llama vs. pork) used as raw material, *L. sakei*, *L. plantarum*, *S. saprophyticus*, *S. xylosus* and *S. equorum* were the dominant species in both the Italian and Argentinean sausages. Table 2 summarizes the phenotypic and genotypic data obtained for the 205 strains analyzed herein.

Overall, phenotypic tetracycline resistance (TET-R) had a higher incidence (43.9%) than erythromycin resistance (ERY-R) (21,5%) in the 205 analyzed strains, mainly observed in Italian pork sausages (Table 2). Particularly, among *L. sakei* and *L. plantarum* strains, 32 out of 121 (26.4%) were TET-R (only four strains from Argentinean sausages) and 13 strains (10.7%) were ERY-R (all of them isolated from Italian salami). Considering the different species, *L*. *plantarum* showed higher TET-R and ERY-R incidence (45.8% and 25%, respectively) than *L. sakei* (21.6% and 7.2%, respectively). These results are in accordance with those reviewed by Fraqueza [1] regarding the AR of LAB isolated from dry fermented sausages.

As far as the 84 CNS strains are concerned, 58 strains were TET-R (69%) and 31 were ERY-R (36.9%) with higher incidence in Italian sausages for both phenotypes. *S. xylosus* was the most TET resistant species (88%), followed by *S. saprophyticus* (67.8%) and *S. equorum* (54.8%); on the contrary, 51.6% of *S. equorum* was ERY-R followed by *S. saprophyticus* (32.1%) and *S. xylosus* (24%). Similar AR incidence in CNS strains isolated from meat products and commodities was previously reported [14,16,17,30,31,32,33]. Even et al. [31] confirmed that the incidence of TET-R and ERY-R among CNS is species-dependent. 

Regarding to the genotype analysis, *tet*K was the most widespread gene among the 205 strains, followed by *tet*M, *erm*B and *erm*C (Table 2). Similarly, Garofalo et al. [34] reported that *tet*M and *tet*K were the prevalent genes for tetracycline resistance, while a high frequency was found for the *erm*B and *erm*C genes, conferring erythromycin resistance to the pork meat microbiota. 

In accordance with Rebecchi et al. [17], the *tet*K gene was widespread among CNS isolated from Italian pork sausages, while only one *S. xylosus* strain from the llama product harbored the *tet*M determinant. This latter gene was prevalent among *L. sakei* and *L. plantarum* isolates, while *tet*S/*tet*W were only detected in *L. plantarum* from Italian salami. The *erm*B gene was predominant in LAB, particularly in *L. sakei,* while *erm*C was frequently detected among CNS, mainly in *S. equorum*.

In this study, selected genes conferring resistance to tetracycline and erythromycin were investigated since they represented some of the most widely distributed among food lactobacilli and CNS [2,16,17,35]. In this sense, discrepancies between the phenotype and genotype profiles were observed from our results: 21.1% of tetracycline and 52.3% of erythromycin-resistant strains did not harbor the respective genetic determinants investigated in this study (Table 2). Among lactobacilli, more discrepancies were observed regarding to tetracycline phenotype/genotype, while a high number of ERY-R CNS resulted as genetically susceptible. This discordance between the phenotype and genotype was already reported by other authors [18,31,36] and may be explained by the presence of additional genes conferring resistance different than those investigated herein [20,34,37]. For example, many genes conferring tetracycline resistance were described, and among these, *tet*O, *tet*Q, *tet*36, *tet*Z, *tet*O/W/32/O/W/O, *tet*W/O genes were also identified among lactobacilli [38]. With regard to erythromycin resistance, especially among CNS, two main resistance mechanism were identified: ribosomal binding site modification mediated by family of *erm* genes (*erm*A, *erm*B, *erm*C, *erm*Y, and *erm*F) and macrolide efflux mediated by *mef*A, *msr*A/B genes [37,39,40]. 

In contrast to our findings, a susceptible phenotype but resistant genotype have been also reported [41,42] and could be due to the low levels and down regulation of gene expression or by an inactive gene product (a mechanism known as silent genes) [36,42]

### 3.2. Correlation between Phenotype and Genotype

**Figure 1:**
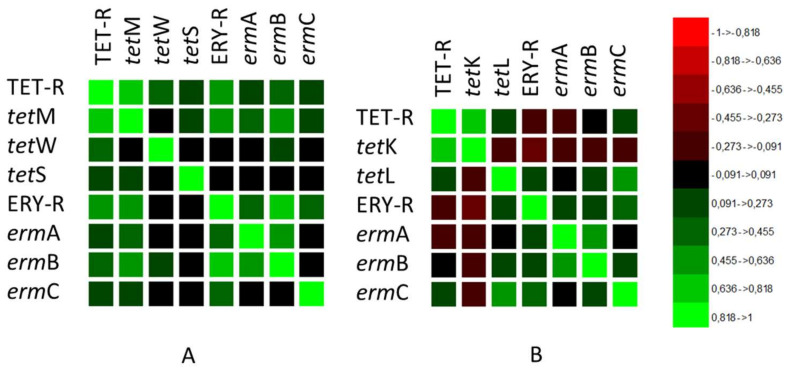
Correlation matrix of phenotypic and genotypic features regarding tetracycline and erythromycin antibiotic resistance in (**A**) LAB species and (**B**) CNS species.

A high correlation (Pearson’s correlation coefficient PC = 0.659) between phenotypic TET-R and ERY-R and genotypic features was observed within the LAB group (Figure 1A). The phenotype TET-R in LAB was highly correlated with the *tet*M gene (PC = 0.743), and ERY-R was highly correlated with the *erm*B gene (PC = 0.808), followed by *erm*A and *erm*C genes with significant correlation (PC = 0.505) (Figure 1A). The TET-R and ERY-R associations and the corresponding genetic determinants are the most frequently described in LAB [6,13,43,44,45,46]. In fact, several studies have reported that TET and ERY resistance genes are highly transferable due to their association with known transposable elements [35,47,48]. 

In the CNS group (Figure 1B), contrarily to pathogenic staphylococci such as *S. aureus* [49], no correlation between the TET-R and ERY-R phenotypes was observed (PC = −0.092). TET-R was positively correlated with the occurrence of the *tet*K gene (PC = 0.702), while ERY-R was correlated with the presence of the *erm*C gene (PC = 0.405) followed by the *erm*B gene (PC = 0.258). The high prevalence of *tet*K related to tetracycline resistance in CNS was reported by other authors [14,31,32,33]. In addition to *tet*K and other AR genes such as *bla*Z, *tet*M, *lnu*A, *erm*B and *erm*C may be located on transferable plasmids; therefore antibiotic-resistant CNS can act as a reservoir and vehicle for human bacteria, representing an important hazard in fermented food [31,50]. 

### 3.3. Correlation between the Tetracycline and Erythromycin MICs Values and Their Genetic Determinants 

The correlation between the MIC values and the genetic determinant incidence was investigated. For the LAB strains, both the MICs and the presence of genes were strictly strain dependent, as shown in Table 3. The Chi-square test (df = 15) = 46.53, *p* < 0.0001) and Pearson’s contingency coefficient (PC = 0.77) demonstrated a strong association between the high MICs and the presence of at least one genetic determinant among the TET-R LAB strains. 

In accordance with the data reported by other authors [6,13,51], we observed that the presence of the *tet*M gene was associated with different MIC values (32–256 µg/mL) among the lactobacilli strains evaluated. Comunian et al. [13] reported that increasing *tet*M transcript levels were correlated with increasing MIC values, suggesting that the expression of this gene is tetracycline concentration dependent. Furthermore, Ammor et al. [52] observed that the presence of more than one tetracycline resistance gene resulted in elevated MICs. In this study, the highest MIC value (512 µg/mL) was observed in two *L*. *plantarum* strains, one harboring *tet*M and *tet*S and one carrying only *tet*W (Table 3). 

Additionally, for the phenotype ERY-R in lactobacilli, there was an association between high MICs (>16 µg/mL) and the occurrence of at least one genetic determinant (*p* < 0.0630; Pearson’s contingency coefficient = 0.78). Strains with lower MICs (4 and 16 µg/mL) were negative for the genetic determinants investigated herein. As stated above and detailed in Table 3, all lactobacilli strains that were ERY-R were also TET-R. For the ERY-R strains, only the presence of *ermB* resulted in elevated MICs (32–512 µg/mL) among lactobacilli strains. The simultaneous occurrence of *erm*A and *erm*B was observed in only two *L. sakei* strains with MICs of 32 and 128 µg/mL, respectively, while *erm*C was detected in only one *L. plantarum* strain with an MIC of 128 µg/mL (Table 3).

In the CNS group (Table 4), resistance to both tetracycline and to erythromycin was observed with MICs ≥ 32 µg/mL. Unlike lactobacilli, no association was observed between higher MICs and the presence of the genetic determinants here investigated herein (Chi-square of Pearson (df = 12) = 8.94, *p* = 0.7084; Pearson’s contingency coefficient = 0.37 and Chi-square of Pearson (df = 12) = 10.87, *p* = 0.5399; and Pearson’s contingency coefficient = 0.52, respectively). In fact, nine strains displaying higher tetracycline MIC (≥128 µg/mL) and 16 strains with erythromycin MIC ≥ 64 µg/mL were negative for all the genes analyzed in this study (Table 4). Among the TET-R CNS carrying the *tet*K gene, 77.6% showed MICs ranging from 32 µg/mL to 512 µg/mL; the co-occurrence of *tet*K and *tet*L was only observed in two *S. xylosus* strains displaying an MIC of 128 µg/mL. The presence of only *tet*L was detected in two strains displaying a high tetracycline MIC (256 µg/mL). Our results indicated that the presence of more than one genetic determinant for tetracycline did not determine higher MICs. The same behavior was observed for one *S. equorum* strain with erythromycin resistance, carrying *erm*A and *erm*B (MIC of 32 µg/mL). The seven CNS strains harboring the *erm*C gene were associated with high erythromycin MIC values (256 µg/mL). Even if CNS may represent a potential safety hazard because some strains are multi-resistant to antibiotics, others display a complete lack of AR, indicating that the AR is strictly strain dependent [31,33,50,53].

### 3.4. LAB and CNS AR Phenotypes: Association with Country of Origin and Manufacturing Practices

**Figure 2:**
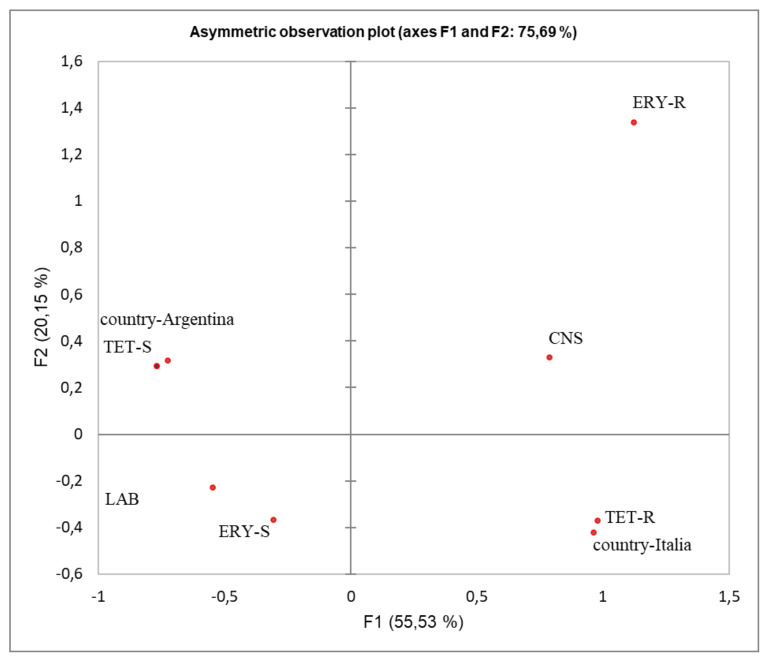
Multivariate correspondence analysis (MCA) summarizing the associations between antibiotic resistance phenotypes (R and S) regarding the LAB and CNS groups isolated from Italian and Argentinean fermented sausages. The contribution to the Chi-squared test is indicated for each axis.

Considering the diverse breeding and manufacturing conditions in Italy and Argentina (intensive vs. extensive practices and the meat production environment, etc.), a multiple correspondence analysis (MCA) was performed to determine the relationships between the country of origin and the TET-R and ERY-R phenotype. The first analysis in Figure 2 clearly distinguished the AR phenotypes (TET-R and ERY-R) associated with the genus *Staphylococcus* belonging to Italian salami from those susceptible of being directly associated with LAB from the Argentinean sausages (TET-S and ERY-S).

In fact, all LAB strains isolated from Argentinean sausages were susceptible to ERY and only four *L. sakei* strains (10%) were TET-R.

**Figure 3:**
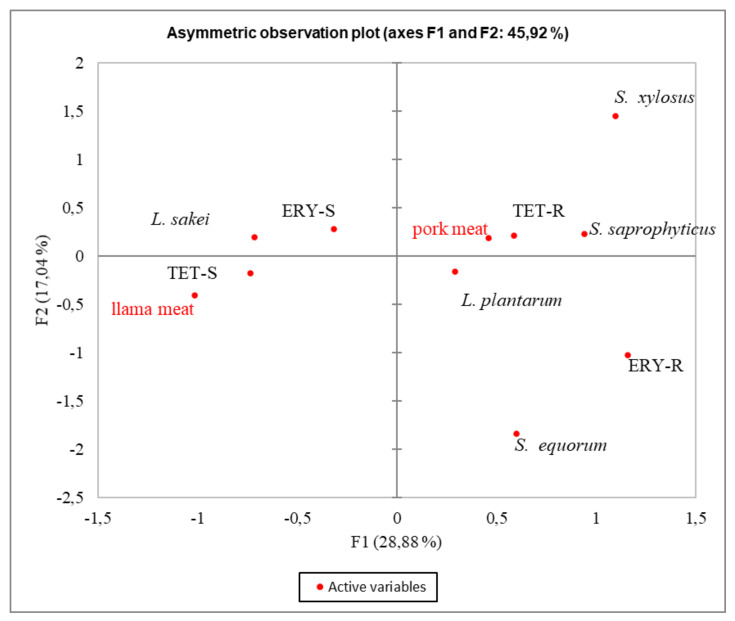
Multivariate correspondence analysis (MCA) summarizing the associations between antibiotic resistance phenotypes (R and S) among *Latilactobacillus* (*L*.) *sakei*, *Lactiplantibacillus* (*L*.) *plantarum*, and *Staphylococcus* (*S*) species isolated from pork meat and llama meat fermented products. The contribution to the Chi-squared test is indicated for each axis.

Additionally, when the different type of meat used and the bacterial species were considered (Figure 3), both AR phenotypes were associated with pork meat, mainly of Italian origin (Figure 2), where intensive practices were used. Moreover, the analysis showed that the TET-R phenotype was associated with *S*. *xylosus* and to a lesser extent, with *S. saprophyticus,* while ERY-R was associated with *L. plantarum* and *S. equorum*. On the contrary, the TET-S and ERY-S phenotypes were correlated with *L. sakei* from llama meat. 

From the MCA, considering either the geographical location or type of meat used, we observed that the AR phenotype of the analyzed strains was associated with Italy and pork meat. Since the Italian fermented products considered in this study were produced using pork meat from intensive farming, we could assume that the strong association observed would be related to farm practices.

To date, very few studies have investigated the frequency of phenotypic antibiotic resistance in the native microbiota of fermented meat products in relation to manufacturing practices. 

The emergence and spread of antibiotic resistance associated with the use of antibiotics in livestock are very complex phenomena with multifaceted effects not only associated with a limited or specific geographical area but which rather depend on farming management [54,55]. The worldwide antibiotics’ misuse in the animal farm, particularly in intensive breeding, has promoted a spread of foodborne AR bacteria. Even though there has been an attempt to control the abusive misuse of antibiotics worldwide (since 2006, the European Union has officially banned the use of antibiotics as feed additives), human “intensive activities”, along with their negative environmental impacts, have contributed to expanding the environmental reservoirs of AR genes [56].

The results obtained in our study demonstrated that the farming practices and the food production context (extensive areas, small food facilities with less negative environmental impact) could influence the different AR occurrence in meat-associated bacteria rather than the different geographical areas considered (Argentina and Italy). Similarly, Haskell et al. [57] reported that the lower rates of antibiotic resistance for *Staphylococcus aureus* found in antibiotic-free animals could apply to other bacterial species. Additionally, antibiotic resistance levels in *Enterococcus* species significantly decreased in a poultry farm in the USA which transitioned from common antibiotic use to organic practices [58]. In addition, Comunian et al. [13] detected the highest number of *Lacticaseibacillus paracasei* resistant strains in fermented meat foods produced in Italian geographical regions where more intensive practices are applied in animal farming.

## 4. Conclusions

In the current study, 205 strains belonging to the LAB and CNS groups isolated from Argentinean and Italian sausages were phenotypically characterized for their resistance to tetracycline and erythromycin, as well as for the presence of their genetic determinants. A higher incidence of TET-R compared to ERY-R was found among the analyzed strains. All lactobacilli strains which were ERY-R were also TET-R, showing a strong association between the high MIC values and the presence of at least one genetic determinant. Regarding the resistant CNS strains, despite the high MIC values observed, no association with the presence of the genetic determinant investigated was found. 

Data analysis highlighted the association between the AR phenotypes and *Staphylococcus* species isolated from Italian salami, while the susceptible phenotypes (TET-S and ERY-S) were associated with the lactobacilli from pork and llama Argentinean sausages. Our findings suggest that TET-R and ERY-R are probably related to certain animal breeding and manufacturing practices rather than the type of meat.

Since animal production systems are not closed ecosystems, antibiotic resistance in meat-associated bacteria is a very complex phenomenon possessing multifaceted effects which is not only associated with a limited specific geographical area but is also significantly affected by different human activities in the environment. The assessment of bacterial resistance in different environmental contexts will help in monitoring the impact of different farming practices and the food production chain on the incidence and spread of antibiotic resistance.

## Figures and Tables

**Table 1:** Antibiotics used, range of concentrations (µg/mL) for MIC determination, and breakpoint values.

Antibiotic	Range of Concentration (µg/mL)		MIC Breakpoint (µg/mL)		
	LAB	CNS	*L. sakei*	*L. plantarum*	CNS
Tetracycline	4–512	4–256	>8	>32	≥16
Erythromycin	0.25–512	4–256	>1	>1	≥8

*L. sakei* and *L. plantarum*: MIC breakpoint [27] CNS: MIC breakpoint [25].

**Table 2:** *Latilactobacillus* (*L.*) *sakei, Lactiplantibacillus* (*L.*) *plantarum* and *Staphylococcus* (*S.*) strains included in this study. Incidence, phenotypic AR and molecular identification of antibiotic-resistant genes.

Meat Products	Species	N° of Isolated Strains	Phenotypic AR		Genotypic AR							
			TET	ERY	*tet*M	*tet*K	*tet*W	*tet*S	*tet*L	*erm*A	*erm*B	*erm*C
Italian pork sausages	*L. sakei*	24	17	7	11	-	-	-	-	2	5	-
	*L. plantarum*	12	11	6	5	-	4	1	-	-	3	1
	*S. xylosus*	22	21	5	-	18	-	-	3	-	1	2
	*S. equorum*	19	16	11	-	10	-	-	1	-	1	4
	*S. saprophyticus*	11	10	3	-	9	-	-	-	-	-	1
Argentinean pork sausages	*L. sakei*	33	4	0	-	-	-	-	-	-	-	-
	*L. plantarum*	7	0	0	-	-	-	-	-	-	-	-
	*S. xylosus*	1	0	1	-	-	-	-	-	-	-	-
	*S. equorum*	2	1	2	-	1	-	-	-	1	1	
	*S. saprophyticus*	10	5	4	-	2	-	-	-	-	-	-
Argentinean llama sausages	*L. sakei*	40	0	0	-	-	-	-	-	-	-	-
	*L. plantarum*	5	0	0	-	-	-	-	-	-	-	-
	*S. xylosus*	2	1	0	1	1	-	-	-	-	-	-
	*S. equorum*	10	0	3	-	-	-	-	-	-	-	-
	*S. saprophyticus*	7	4	2	-	4	-	-	-	-	-	-
		205	90	44	17	45	4	1	4	3	11	7
	AR incidence %		43.9	21.5	8.293	21.95	1.951	0.488	1.95	0.488	5.366	3.415

**Table 3:** Tetracycline (TET-R) and erythromycin (ERY-R)-resistant LAB species: *Latilactobacillus* (*L.*) *sakei and Lactiplantibacillus* (*L.*) *plantarum*. MIC values measured by broth microdilution method using LSM (μg/mL) and the distribution of genetic determinants investigated in this study.

LAB		TET-R	Genetic Determinant					ERY-R	Genetic Determinant		
		MIC	*tet*M	*tet*K	*tet*W	*tet*S	*tet*L	MIC	*erm*A	*erm*B	*erm*C
*L. sakei*	UC103	64									
	UC109	32						4			
	UC12	16									
	UC128	128						4			
	UC13	128						128			
	UC131	256						256			
	UC133	64									
	UC136	64									
	UC17	16									
	UC23	16						32			
	UC32	32									
	UC35	16									
	UC38	16									
	UC49	64						32			
	UC57	64									
	UC65	64									
	UC89	256						128			
	UC9096	16									
	UC9097	16									
	UC8437	16									
	UC8438	16									
*L. plantarum*	UC108	256									
	UC125	256						512			
	UC126	256						256			
	UC129	256						128			
	UC144	64									
	UC145	64									
	UC146	256						256			
	UC2	512									
	UC24	512									
	UC25	128						4			
	UC7	64						4			

Positive for the antibiotic resistance genetic determinant. 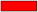 * MICs for TET (μg/mL): 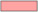 16–32; 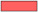 64–128; 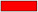 256–512. * MICs for ERY (μg/mL): 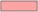 4–16; 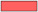 32–64; 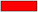 256–512.

**Table 4:** Tetracycline- (TET-R) and erythromycin- (ERY-R) resistant *Staphylococcus* strains. MIC values were measured by broth microdilution method using CAMHB (μg/mL) and the distribution of the genetic determinants investigated in this study.

*Staphylococcus (S)*		TET-R	Genetic Determinants					ERY-R	Genetic Determinants		
		*MIC	*tet*M	*tet*K	*tet*W	*tet*S	*tet*L	*MIC	*erm*A	*erm*B	*erm*C
*S. saprophyticus*	UC8721							32			
	UC8722							64			
	UC8723	32									
	UC8724	32									
	UC8738	32									
	UC8731	32						64			
	UC8736	32									
	UC7548							64			
	UC7557	64									
	UC7547	64									
	UC7546	64									
	UC7545	64									
	UC117	256									
	UC905	128						256			
	UC929	256						128			
	UC705	128									
	UC51	128									
	UC732	128									
	UC879	256						256			
	UC797	256									
	UC114	128									
	UC111	256									
*S. xylosus*	UC7543	64									
	UC8727							32			
	UC662	256									
	UC666	128									
	UC741	512									
	UC761	256									
	UC852	256									
	UC862	256						256			
	UC132	256						256			
	UC756	512									
	UC776	512						128			
	UC109	128									
	UC101	128									
	UC789	256									
	UC897	256						256			
	UC120	128									
	UC48	128									
	UC113	128									
	UC119	128									
	UC115	128									
	UC431	128									
	UC432	128						256			
	UC784	512									
*S. equorum*	UC8728							32			
	UC8729	32									
	UC7559							64			
	UC7568							32			
	UC7569							64			
	UC773	256									
	UC133	5						128			
	UC79	128						256			
	UC917	512						256			
	UC740	256						256			
	UC753	256						256			
	UC638	128						256			
	UC105	256									
	UC123	256									
	UC786	256						128			
	UC829	256									
	UC823							128			
	UC934							256			
	UC629							256			
	UC127	128						256			
	UC121	256									
	UC122	256									
	UC832	256									
	UC102	256									

Positive for the antibiotic resistance genetic determinant 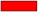 * MICs for TET and ERY (μg/mL) 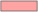 16–32; 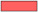 64–128; 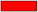 256–512.

## Data Availability

Not applicable.

## Supplementary Materials

The following are available online at https://www.mdpi.com/article/10.3390/microorganisms9102111/s1, Table S1. Primer used in this study.
