# Supplementary material for: Incidence of Tetracycline and Erythromycin Resistance in Meat-Associated Bacteria: Impact of Different Livestock Management Strategies
Source: Microorganisms. 2021 Oct 7;9(10):2111. doi: 10.3390/microorganisms9102111 (PMC8537249; doi:10.3390/microorganisms9102111)
Supplement: Supplementary file 1 [file microorganisms-09-02111-s001.zip › microorganisms-1392004-supplementary.pdf]

Table S1. Primer used in this study.

Target gene	Forward primer (5'- 3')	Reverse primer (5'- 3')	Product (bp)	References
tetK	TATTTTGGCTTTGTATTCTTTCAT	GCTATACCTGTTCCCTCTGATAA	1159	Trzcinski et al., 2000
tetL	ATAAATTGTTTCGGGTCGGTAAT	AACCAGCCAACTAATGACAATGAT	1077	Trzcinski et al., 2000
tetM	GAACTCGAACAAGAGGAAAGC	ATGGAAGCCCAGAAAGGAT	740	Olsvik et al., 1995
*tet*W	GAGAGCCTGCTATATGCCAGC	GGGCGTATCCACAATGTTAAC	168	Aminov et al. 2001
tetS	GAAAGCTTACTATACAGTAGC	AGGAGTATCTACAATATTTAC	169	Aminov et al. 2001
ermA	TCTAAAAAGCATGTAAAAGAA	CTTCGATAGTTTATTAATAATAGT	645	Sutcliffe et al., 1996
ermB	GAAAAGGTACTCAACCAAATA	AGTAACGGTACTTAAATTGTTTAC	639	Sutcliffe et al., 1996
ermC	TCAAAACATAATATAGATAAA	GCTAATATTGTTTAAATCGTCAAT	642	Sutcliffe et al., 1996
